# Oxidative Stress as a Therapeutic Target of Cardiac Remodeling

**DOI:** 10.3390/antiox11122371

**Published:** 2022-11-30

**Authors:** Danilo Martins, Leonardo Rufino Garcia, Diego Aparecido Rios Queiroz, Taline Lazzarin, Carolina Rodrigues Tonon, Paola da Silva Balin, Bertha Furlan Polegato, Sergio Alberto Rupp de Paiva, Paula Schmidt Azevedo, Marcos Ferreira Minicucci, Leonardo Zornoff

**Affiliations:** 1 Internal Medicine Department, Botucatu Medical School, São Paulo State University (UNESP), Botucatu 01049-010, Brazil; 2 Surgery and Orthopedics Department, Botucatu Medical School, São Paulo State University (UNESP), Botucatu 01049-010, Brazil

**Keywords:** antioxidants, heart failure, cardiac remodeling

## Abstract

Cardiac remodeling is defined as a group of molecular, cellular, and interstitial changes that clinically manifest as changes in the heart’s size, mass, geometry, and function after different stimuli. It is important to emphasize that remodeling plays a pathophysiological role in the onset and progression of ventricular dysfunction and subsequent heart failure. Therefore, strategies to mitigate this process are critical. Different factors, including neurohormonal activation, can regulate the remodeling process and increase cell death, alterations in contractile and regulatory proteins, alterations in energy metabolism, changes in genomics, inflammation, changes in calcium transit, metalloproteases activation, fibrosis, alterations in matricellular proteins, and changes in left ventricular geometry, among other mechanisms. More recently, the role of reactive oxygen species and oxidative stress as modulators of remodeling has been gaining attention. Therefore, this review assesses the role of oxidative stress as a therapeutic target of cardiac remodeling.

## 1. Introduction

Cardiac remodeling plays a pathophysiological role in the onset and progression of ventricular dysfunction and subsequent heart failure. Therefore, strategies to mitigate this process are critical. Recently, the role of reactive oxygen species and oxidative stress as modulators of remodeling has been gaining attention. Therefore, this review assesses the role of oxidative stress as a therapeutic target of cardiac remodeling.

## 2. Cardiac Remodeling

Cardiac remodeling is defined as molecular, cellular, and interstitial changes manifested clinically as alterations in the heart’s size, shape, and function after different stimuli [1].

It is worth highlighting the differentiation between physiological (compensatory) cardiac remodeling and pathological (maladaptive) remodeling. However, this review will address aspects related only to pathological remodeling.

The important point is that numerous injury triggers are recognized, including cardiac infarction, hypertension, diabetes, valvular diseases, toxicity, inflammation, arrhythmia, genetic cardiopathies, and other conditions [1]. It is important to emphasize that the main cellular element altered in remodeling is the cardiomyocyte. Still, other cells are involved in the process, such as fibroblasts, immune system cells, and coronary vasculature cells [2].

Another relevant issue is that cardiac remodeling may result in progressive ventricular dysfunction, the clinical presentation of heart failure, and cardiovascular death. Therefore, reversing or preventing further remodeling is critical to improving the prognosis associated with different cardiac injuries.

**Figure 1:**
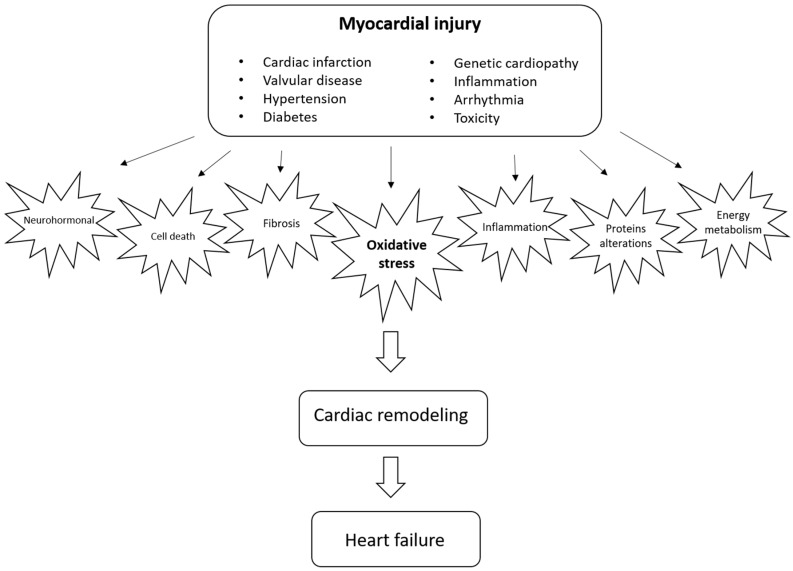
Natural history of cardiac remodeling.

Several pathways can modulate cardiac remodeling, including neurohormonal activation, increase in cell death, alterations in contractile and regulatory proteins, alterations in energy metabolism, changes in genomics, inflammation, changes in calcium transit, metalloprotease activation, fibrosis, alterations in matricellular proteins, and changes in left ventricular geometry, among other mechanisms [1,3]. In recent years, however, due to its relevance and therapeutic potential, oxidative stress has gained prominence as a critical modulator of cardiac remodeling [4], as shown in Figure 1.

## 3. Oxidative Stress as a Potential Modulator of Cardiac Remodeling

Oxidative stress is the disproportion between the formation of reactive oxygen species (ROS) and the inactivation capacity of the cellular endogenous antioxidant system. It may result from increased production of ROS and dysfunctions of the antioxidant defense [5]. The balance between ROS production and its inactivation is recognized as the redox state [6].

Reactive oxygenated or nitrogenated species (ROS/RNS) include free radicals and nonradicals with high reactivity. They are represented by hydrogen peroxide, superoxide anion radical, singlet oxygen, and hydroxyl radical [6,7]. Reactive nitrogen species, such as nitric oxide, peroxynitrite, iron, copper, and sulfur, are also encountered [8,9]. The biochemical interaction between ROS and RNS, products of oxidative stress, with nucleic acids, lipids, and proteins, determines structural changes and functions responsible for several pathological processes, including cardiovascular disease and cardiac remodeling. Despite the widely described deleterious biological effects of ROS and RNS, they are also responsible for other processes related to homeostasis, such as participation in cellular immune defense, cell signaling, and biosynthetic reactions, for example [10].

Superoxide radical anion results from a one-electron reduction of oxygen by oxidases, like nicotinamide adenine dinucleotide phosphate oxidase (NADPH oxidase or NOX), xanthine oxidase (XO), and cyclooxygenase (COX). It can also be formed in the mitochondrial electron transport chain during oxidative phosphorylation responsible for ATP formation [11]. Superoxide anion is an active nucleophile that can react to positively charged centers and dismutate, yielding molecular oxygen and hydrogen peroxide [7].

Hydrogen peroxide can be generated from superoxide and in the presence of oxidases (urate oxidase, glucose oxidase, D-amino acid oxidase) which catalyze two-electron transfer to molecular oxygen. Hydrogen peroxide can produce highly reactive radicals by interacting with hemeprotein structure with an iron release, enzyme inactivation, and oxidation of DNA, lipids, sulfur groups, and keto acids. It is converted to water by catalase, a ferriheme-containing enzyme [7].

On the other hand, the hydroxyl radical is responsible for the oxidative damage due to its aggressiveness. It results from Fenton-type reactions and radiolysis of water [12]. This radical is the most powerful oxidizing and can interact with DNA, proteins, lipids, amino acids, sugars, and metals [7]. Molecular oxygen has oxidative power due to the remotion of spin restriction of oxygen [12].

Another important reactive species is peroxynitrite. It is formed by the reaction of superoxide anion radical and endogenous nitric oxide (NO) [13], involved in vasodilation and neurotransmission. NO is synthesized from L-arginine, oxygen, and NADPH by enzymes belonging to the nitric oxide synthase (NOS) class [7]. Peroxynitrite, in turn, is a very reactive and damaging nitrogen species and a powerful oxidant to many biomolecules. Peroxynitrite can be decomposed to hydroxyl radicals, and its protonated form can act as depleting sulfhydryl groups, causing oxidative derangement on several biomolecules like hydroxyl radicals. It also can cause DNA damage through breaks, protein oxidation, and nitration of aromatic amino acid moieties in protein structure [7].

Under physiological situations, superoxide anion and hydrogen peroxide are not very reactive, and hydroperoxides proved almost stable. Their reactivity is enhanced by the presence of hemeproteins and low molecular weight transition metal chelates, which may produce more reactive species such as hydroxyl radical or ferryl hemeprotein radical. The latter participates in the formation of alkoxyl and peroxyl radicals. However, suppose the antioxidant system does not act at the proper sites. In that case, oxidative damage of enzymes, ion channels, structural proteins, and membrane lipids may occur, impairing many cell functions, bringing pathological consequences, and even cell death [7]. For example, global ischemia in the heart leads to a 250% increase in reactive oxygen species [14]. In this way, the presence of ROS and RNS in pathological status and not properly counterbalanced by the antioxidant defenses is strictly related to cytotoxicity, which can explain, at a molecular level, the occurrence of cardiac remodeling and cardiac dysfunction.

The deleterious action of ROS is directly involved in the pathogenesis of cardiac tissues. They promote myocardial growth, extracellular matrix remodeling, and cellular dysfunction by activating hypertrophy signaling kinases and transcription factors [6].

Stimuli such as angiotensin II, bradykinins, endothelins, α-adrenergic stimulation, and ROS act on cardiac G-protein [15]. After the described stimulation, hypertrophic signaling pathways are activated [16]. It is also well known that G-protein coupled receptor activation can lead to ROS generation [5]. Thus, ROS, by stimulating G-protein and being a by-product of its action, promote hypertrophic growth signaling in ventricular myocytes [15].

ROS also stimulates cellular apoptosis, signaling kinase-1, a redox-sensitive kinase, which, when overexpressed, leads to nuclear factor kappa beta (NF-ĸB)-induced hypertrophy by specifically related gene expression [6,15].

Inside the cardiomyocyte nucleus, ROS may cause DNA strand breaks, activating the enzyme poly (ADP-ribose) polymerase-1 (PARP-1), which then regulates the expression of a variety of inflammatory mediators involved in cardiac remodeling progression [6].

The previously described hydrogen peroxide, an important ROS, can activate a few kinase signaling pathways related to hypertrophy and apoptosis. These effects are mediated by extracellular signal-regulated kinase 1/2 (ERK 1/2), c-Jun-N-terminal kinase (JNK), p38 mitogen-activated protein kinase, protein kinase B (Akt), and mitogen-activated protein kinase (MAPK). Akt, MAPK, JNK, and p38 mitogen-activated protein kinase are involved in apoptosis, and ERK 1/2 is related to hypertrophy.

The extracellular matrix (ECM) is another potential target for the deleterious action of ROS. ECM comprises a well-organized function network of macromolecules that provides structural support for the cells and tissues and with a significant role in health and disease [17]. ROS can affect ECM by stimulating the proliferation of cardiac fibroblasts [15], resulting in fibrosis and matrix remodeling. Another described interaction is based on ROS action activation matrix metalloproteinases (MMPs) [6]. They also stimulate transcription factors such as NF-ĸB and activator protein-1 to activate MMP expression [5]. Furthermore, MMPs play a pivotal role in normal tissue remodeling processes, such as cell migration, invasion, proliferation, and apoptosis [17], and have been demonstrated to be elevated in failing hearts [6], which makes them the target of potential therapeutic interventions [18].

Oxidative stress is also an important driving force, leading to changes in the cellular cytoskeleton that accompany ventricular remodeling [19]. As part of the cellular cytoskeleton apparatus, microtubules are cylindrical polymers of α/β-tubulin heterodimers that form a complex network throughout the cytoplasm [20]. Oxidative stress then leads to cysteine oxidation of microtubules, and GTP-tubulin incorporation into these damaged and oxidized sites facilitates a pathogenic shift from a sparse microtubule network into a dense, aligned network with a remarkable cellular contractile failure [14].

Another detrimental effect carried out by excessive ROS occurs in ryanodine receptors (RyRs), localized to the sarcoplasmic reticulum (SR) [21]. They are responsible for calcium release from SR during excitation–contraction coupling in striated muscle cells [22].

ROS promotes RyRs activity and inhibits SR calcium-adenosine triphosphatase 2 (SERCA-2) activity, resulting in calcium overload and reduced myofilament calcium sensitivity, leading to contractile dysfunction [21]. Most studies indicate that the oxidation of thiol (SH) groups of RyRs is directly responsible for ROS’s effects [23]. It has been demonstrated that the open probability of cardiac muscle RyRs is increased in the presence of superoxide, hydrogen peroxide, and hydroxyl radical [24]. Data indicate ROS can irreversibly inactivate RyRs with calcium overload and contractile dysfunction [25].

Mitochondria, the cell powerhouse, is a key point to understanding the interface between ROS and cardiac failure and remodeling. These organelles play an important role in maintaining cellular redox balance. Electron transport chain complexes, in charge of ATP synthesis, are a major source of ROS which then are counterbalanced by antioxidant defense systems [5]. Indeed, mitochondrial or antioxidant protein disturbances have been associated with cardiac hypertrophy. ROS can also affect ATP levels, as evidenced by a study that related the status of energy metabolism in myocardial infarction with significantly impaired ATP levels [26]. In this way, mitochondria react to ischemic injury by producing increased levels of ROS, leading to a perverse cycle of energy metabolism and mitochondrial dysfunction [21].

Besides being a relevant source of ROS, mitochondria can also be affected by them, with macromolecules damaged at or near the site of their formation [6]. This organelle has its genomic system, mitochondrial DNA (mtDNA), a closed circular double-stranded molecule responsible for controlling mitochondrial function with factors that regulate its transcription and replication [27]. In turn, failing hearts have increased ROS generation associated with mitochondrial damage and dysfunction. This might be explained by some details related to the organelle itself.

Some features of mitochondria make them more susceptible to oxidative stress. They do not have a complex chromatin organization with histone proteins, which act as defense barriers against ROS, they have a limited repair activity against mtDNA damage, and a large part of superoxide formed inside mitochondria cannot pass through the membranes, causing significant functional impairment [6]. ROS then are involved in a deleterious cycle of functional mitochondrial decline, subsequential ROS generation, and cellular injury taking place in important clinical conditions such as post-myocardial infarction, one of the leading causes of cardiovascular diseases, heart failure and cardiac remodeling [28].

More evidence of the association between oxidative stress and cardiac remodeling was provided by an experimental study conducted by Li et al. that evaluated the activity of NADPH oxidase, one of the major sources of ROS production, in an experimental guinea pig model of left ventricular (LV) hypertrophy due to progressive pressure overload [29]. During the development of cardiac hypertrophy, data revealed increasing NADPH-dependent ROS and protein expression of NADPH oxidase subunits, specifically p22^phox^, gp91^phox^, p67^phox^, and p47^phox^. Further, activation of redox-sensitive singling pathways occurred in parallel to the ROS production, as demonstrated by increased levels of phosphorylated ERK 1/2, ERK 5, p38 MAPK, and JNK. Another experimental study with cardiac remodeling induced in farm pigs by volume overload showed similar results in oxidative stress biomarkers [30]. Levels of malonaldehyde (MDA) and 4-hydroxyalkenals (4-HNE), two products of the lipoperoxidation process, and JNK and ERK phosphorylation increased in an acute stage of volume overload in animal models.

Oxidative stress may occur due to environmental factors like aging, diet, and smoking [31,32,33]. Mayyas et al. evaluated the effect of smoking on cardiac biomarkers of oxidative stress, inflammation, and cardiac fibrosis by using different smoking devices in Male Wistar rats [34]. All exposed rats had an increased heart-to-body ratio and area of cardiac fibrosis compared to the control group. Moreover, superoxide dismutase activity increased in rats exposed to electronic cigarette aerosol and tobacco cigarette smoke, probably in response to the excess ROS. Another variable that may influence the balance between the production of ROS and antioxidants is the diet. Tsutsui et al. showed in an experimental study that Dahl salt-sensitive rats, when fed with a high-salt diet, have increased production of ROS evaluated by electron spin resonance spectroscopy in the myocardium [33]. Hence, rats with a high-salt diet had increased LV weight and mortality compared to those with a low-salt diet.

Considering the mechanisms of oxidative stress involved in the pathophysiology of cardiac remodeling, some biomarkers may have a prognostic value in patients with heart failure [34,35,36,37,38]. A study with 843 patients evaluated 37 biomarkers from different domains, including oxidative stress, in patients with heart failure at hospital admission and after 24 h [36]. Among the biomarkers, changes in myeloperoxidase (MPO) were related to outcomes in heart failure with preserved eject fraction patients. Another biomarker, growth differentiation factor-15 (GDF-15), is a cytokine of the transforming growth factor beta (TGF-β) family that is produced by cardiac myocytes in response to oxidative stress [39]. A Chinese observational study showed that this biomarker is an independent predictor of mortality in patients with heart failure with low ejection fraction [37]. In addition, indoxyl sulfate (IS), a gut-derived uremic toxin, increases endothelial ROS production and decreases glutathione levels in endothelial cells [40]. In an observational study with patients submitted to radiofrequency catheter ablation, IS was an independent predictor of the recurrence of atrial fibrillation (AF) [38].

Despite the association between some markers of oxidative stress and prognosis, not even all markers have the same prediction. In a similar study with patients submitted to radiofrequency catheter ablation for AF, high levels of soluble advanced glycation end product receptors (sRAGE) were an independent predictor of the recurrence of AF only in diabetic patients [41]. The same association was not present in patients without diabetes in the same study. Another recent study with 53 patients analyzed the role of different biomarkers in patients with heart failure [42]. An accurate oxidative stress marker, urinary 8-iso-PGF2α, was not correlated with poor outcomes in the population studied.

Therefore, most evidence suggests that oxidative stress is an important modulator of the cardiac remodeling process in different models.

## 4. Evidence of Oxidative Stress as a Therapeutic Target against Cardiac Remodeling

Given its involvement in cardiac remodeling and heart diseases, oxidative stress may be an attractive target for remodeling therapies. Indeed, studies with different diets and natural food rich in antioxidants have investigated its role in preventing and treating cardiovascular diseases [15]. Recently, three experimental studies evaluated the role of açai, a fruit rich in polyphenols, in different models of cardiovascular disease in rats [43,44,45]. After açai supplementation, myocardial infarction rats had a lower concentration of MDA, an oxidative stress marker, and cardiac remodeling [45]. Similar results were found with açai seed extract in the renovascular hypertension animal model and obese high-fat diet-fed mice, demonstrating a protective effect in cardiovascular diseases probably due to its antioxidant properties [43,44]. Orange juice (*Citrus sinensis*), grape seed extract, and jaboticaba (*Myrciaria jaboticaba*), fruits with antioxidant components, also attenuated cardiac remodeling after myocardial infarction in rats [46,47,48].

Positive results with natural feed led to investigation of antioxidant components derivative with potential benefits in cardiovascular diseases. Indeed, Resveratrol, a polyphenol present in different plants such as grapes and berries may also have beneficial effects in preventing cardiac fibrosis due to heart diseases, as was especially demonstrated in experimental models [49].

Glutathione, a tripeptide with complex cellular functions, including the ability to reduce ROS, is another target for developing therapies for heart diseases [50]. A recent clinical trial evaluated glutathione compared to a placebo in patients with ST-segment-elevation myocardial infarction and submitted to primary percutaneous coronary intervention [51]. The intervention led to a significant reduction in soluble NOX2 and an increase in NO activity compared to the placebo. In addition, the glutathione group had decreased left ventricular size and increased cardiac function.

Despite the promising results in experimental models and the plausibility of reducing oxidative stress, either for attenuating ROS production or increasing serum antioxidants, there is still a lack of evidence showing the benefits of treatments focusing on this pathophysiological pathway in clinical trials and even in some experimental models [5,49,52]. In an experimental study, Resveratrol was tested combined with Sacubitril/Valsartan to evaluate its effects on cardiac remodeling in myocardial infarction (MI)-induced rats [53]. The results revealed that the combination of Sacubitril/Valsartan with Resveratrol significantly prevented left ventricular remodeling after induction of MI and reduced MDA levels. However, these results were not significantly beneficial compared to Sacubitril/Valsartan or Valsartan alone. Among clinical trials, there is a lack of studies comparing the effects of Resveratrol with a placebo in heart failure. Two small clinical trials, one in patients with heart failure and the other with stable coronary artery disease, showed potential benefits with Resveratrol compared with a placebo in left ventricular function [54,55]. Despite these benefits, more studies must be conducted to understand the role of Resveratrol in heart failure patients.

L-carnitine, a molecule that participates in cellular defenses against ROS, was also evaluated in a clinical trial involving patients undergoing coronary artery bypass graft surgery [56]. After 180 days, L-carnitine supplementation did not demonstrate additional benefit compared to placebo reverse remodeling despite its antioxidant properties.

Antioxidant supplements were also tested in experimental models and clinical trials. Ascorbic acid (vitamin C), an antioxidant supplement widely available, was tested in clinical trials and experimental models [57,58]. In a doxorubicin-induced cardiomyopathy experimental model, vitamin C improved left ventricular function and reduced thiobarbituric acid-reactive substances (TBARSs) [59]. However, no significant differences in glutathione peroxidase (GPx) and superoxide dismutase (SOD) activities, two important antioxidant enzymes, were found in rats treated with vitamin C after the induced cardiomyopathy. In an experiment with acute Chagas cardiomyopathy induced in Swiss male rats, vitamin C and vitamin E were also tested [60]. Even considering its benefits as an antioxidant therapy, these two supplements could not reduce oxidative stress and inflammation damage due to acute infection by *Trypanosoma cruzi*. A clinical trial with patients undergoing coronary artery bypass graft surgery also did not show a significant difference between vitamin C supplementation and placebo during the postoperative period in the incidence of atrial fibrillation despite the antioxidant function [61]. The study did not evaluate the parameters of ventricular function.

In parallel with the development of new therapies focusing on reducing ROS, existing treatments for cardiovascular diseases have evidence of reducing oxidative stress by different pathways. Dipeptidyl-peptidase 4 (DPP-4) inhibitors and glucagon-like peptide 1 (GLP-1) agonists, two classes of medication for the treatment of type 2 diabetes, were assessed in an experimental model of myocardial ischemia in rats [62]. The study revealed that linagliptin, a DPP-4 inhibitor drug, and liraglutide, a GLP-1 agonist medication, reduced infarct size. Moreover, both treatments promoted a reduction of ROS generation, NADPH oxidase, and collagen deposition. However, an observational study in type 2 diabetes mellitus humans did not show a significant reduction in oxidative stress biomarkers treated with DPP-4 inhibitors [63,64].

Recently, American Heart Association (AHA) recommended sodium-glucose cotransporter 2 (SGLT2) inhibitors as first-line therapy for patients with heart failure, independently of the presence of type 2 diabetes [65]. In addition to the glycosuric effect, SGLT2 inhibitors may play a role in reducing oxidative stress, but the mechanisms are still uncertain [66,67,68]. One proposed mechanism is that SGLT2 inhibitors, by promoting increased glycosuria, reduce urate reabsorption and then reduce plasma uric acid, an agent associated with oxidative stress and inflammatory response [68]. Other studies suggest that gliflozins may reduce NADPH oxidase activity by different pathways. In a genetically modified type 2 diabetes mellitus mice model, empagliflozin reduced oxidative stress by activating the nuclear factor erythroid 2 (Nrf2)/antioxidant responsive element (ARE) pathway and suppressing the transforming growth factor beta (TGFβ)/Smad pathway [69]. A recent clinical trial with canagliflozin in patients undergoing cardiac surgery demonstrated its effects on NADPH oxidase suppression by analysis of myocardial biopsies and superoxide quantification [70].

Like an SGLT2 inhibitor, metformin, another oral drug for type 2 diabetes, may play a role in cardiovascular disease despite the absence of diabetes. A recent randomized controlled trial assigned patients with coronary artery disease, pre-diabetes, or insulin resistance to receiving either metformin or placebo [71]. They concluded that the metformin group had significantly reduced left ventricular mass indexed to height by cardiac magnetic resonance. In addition, the metformin group had reduced levels of thiobarbituric acid reactive substances (TBARs), a biomarker of oxidative stress.

Other potential agents that may become important tools in the treatment of heart failure, due to their antioxidant properties, are guanylate cyclase stimulators, and NO modulators.

NO is synthesized from L-arginine, diffuses rapidly into vessel smooth muscle cells, and catalyzes the conversion of guanosine triphosphate (GTP) into the second intracellular messenger, cGMP. This, in turn, stimulates several transduction cascades that mediate various physiological and protective effects, including smooth muscle relaxation, inhibition of cellular proliferation, and decreased inflammation [72]. This mechanism could explain the beneficial effects of Sacubitril/Valsartan in patients with heart failure and reduced ejection fraction (HFrEF), as Sacubitril would induce an increase in NO levels.

Likewise, Vericiguat is a drug that stimulates the cGMP pathway through stimulation of soluble guanylate cyclase. The VICTORIA trial enrolled 5050 subjects with HFrEF. Patients were randomized to placebo or Vericiguat. The study showed that in patients treated with Vericiguat, the incidence of primary outcomes (heart failure hospitalizations or cardiovascular death) was lower than in patients treated with placebo [73].

Finally, due to the important role of oxidative stress in modulating cardiac remodeling and subsequent ventricular dysfunction, there are several ongoing studies with drugs targeting oxidative stress in the treatment of heart failure, including Tetrahydrobiopterin (a cofactor for NO synthase), Resveratrol, Alpha-Lipoic Acid, N-Acetylcysteine, and Pentoxifylline. The results of these studies, which will be available in the coming years, maybe determine the definitive role of antioxidants in this clinical scenario.

## 5. Conclusions

Oxidative stress plays a critical role in the modulation of cardiac remodeling, as it acts on different mechanisms involved in this process. Several pieces of evidence have shown that several antioxidants can attenuate the process of cardiac remodeling secondary to different injuries, mainly in animal models, by reducing oxidative stress. Thus, considering the relevance of remodeling, antioxidants have the potential to become part of a therapeutic strategy against this important pathological condition. However, evidence for the benefit of antioxidant therapies in clinical trials is sparse. Therefore, more studies are needed for definitive conclusions about the role of oxidative stress as a therapeutic target of cardiac remodeling.
